# Supplementary material for: Selection and Validation of Reference Genes for Gene Expression in Bactericera gobica Loginova under Different Insecticide Stresses
Source: Int J Mol Sci. 2024 Feb 19;25(4):2434. doi: 10.3390/ijms25042434 (PMC10889791; doi:10.3390/ijms25042434)
Supplement: Supplementary file 1 [file ijms-25-02434-s001.zip › ijms-2828721-supplementary.pdf]

Selection and Validation of Reference Genes for Gene Expression in *Bactericera gobica* Loginova Under different Insecticide Stresses

Hongshuang Wei [1], Jingyi Zhang [1], Mengke Yang [1], Yao Li [1], Kun Guo [1], Haili Qiao [1], Rong Xu [1], Sai Liu [1,*], and Changqing Xu [1,*]

1 Institute of Medicinal Plant Development, Chinese Academy of Medical Sciences and Peking Union Med-ical College, Beijing, 100193, China

* Correspondence: cqxu@implad.ac.cn

Table S1. Best BLAST hits for candidate reference genes and target gene identified in the transcriptome database of *Bactericera gobica* adults and nymphs.

Gene name	transcriptome ID	Length	aa	Complete ORF	Description	E value	Identity (%)	Accession
**Reference genes:**								
β-actin	TRINITY_DN1329_c16_g1	1508	337	Y	Actin 1 [*Laodelphax striatellus*]	0	98.67%	AGJ84696.1
EF1α	TRINITY_DN5961_c2_g1	2703	325	Y	elongation factor 1 alpha [*Bactericera cockerelli*]	0	99.03%	ALX81339.1
EF2	TRINITY_DN8489_c0_g3	4520	844	Y	PREDICTED: translation elongation factor 2 [*Neodiprion lecontei*]	0	93.84%	XP_015509932.1
GAPDH	TRINITY_DN1344_c7_g3	1936	332	Y	glyceraldehyde-3-phosphate dehydrogenase [*Bactericera cockerelli*]	0	98.80%	ALX81341.1
α-TUB	TRINITY_DN999_c1_g1	1328	360	3'lost	tubulin alpha-1 chain-like [*Diaphorina citri*]	0	97.78%	XP_008480775.1
β-TUB	TRINITY_DN22746_c0_g1	3072	447	Y	tubulin beta-1 chain [*Diaphorina citri*]	0	99.11%	XP_008480683.1
Ferritin	TRINITY_DN1667_c1_g1	4196	239	Y	ferritin heavy chain-like precursor [*Diaphorina citri*]	3.00E-98	62.35%	NP_001316112.1
GST	TRINITY_DN13130_c0_g2	1495	216	Y	glutathione S-transferase 1-like [*Diaphorina citri*]	2.00E-148	93.98%	XP_026680107.1
AK	TRINITY_DN9312_c5_g1	3070	259	Y	arginine kinase isoform X2 [*Diaphorina citri*]	8.00E-175	91.41%	XP_026680842.1
RPL8	TRINITY_DN7930_c5_g1	1301	200	Y	60S ribosomal protein L8 [*Diaphorina citri*]	1.00E-139	97.50%	XP_008478127.1
RPL10	TRINITY_DN207_c11_g1	1605	219	Y	60S ribosomal protein L10 isoform X4 [*Diaphorina citri*]	2.00E-151	94.52%	XP_008471214.1
RPL32	TRINITY_DN7277_c2_g1	1090	134	Y	60S ribosomal protein L32-like [*Diaphorina citri*]	3.00E-91	99.25%	XP_008468712.1
RPS11	TRINITY_DN35443_c0_g1	670	152	Y	40S ribosomal protein S11 isoform X3	9.00E-105	98.03%	XP_008470793.1

					[*Diaphorina citri*]			
RPS15	TRINITY_DN24856_c0_g1	1046	130	Y	40S ribosomal protein S15Aa [*Diaphorina citri*]	2.00E-88	99.23%	NP_001284622.1
RPS20	TRINITY_DN12526_c3_g1	554	131	Y	40S ribosomal protein S20 [*Diaphorina citri*]	7.00E-83	96.18%	XP_008476677.1
Target gene:								
CYP6a1	TRINITY_DN4781_c0_g1	3341	585	Y	cytochrome P450 CYP6a1 [*Diaphorina citri*]	0	77.49%	QBQ34510.1

Table S2. Mean Ct values ± SE of the studied reference genes in the different samples f*rom Bactericera gobica.*

Gene name	Insecticide treatment	Temperature treatment	Odor stimulation	Developmental stages	Sex	All samples
β-actin	20.64 ± 0.47	17.33 ± 0.00	16.74 ± 0.05	17.22 ± 0.30	17.34 ± 0.00	18.34 ± 0.30
EF1α	20.33 ± 0.26	17.74 ± 0.09	16.73 ± 0.12	17.57 ± 0.29	17.23 ± 0.09	18.37 ± 0.24
EF2	22.14 ± 0.26	20.15 ± 0.09	20.01 ± 0.07	20.17 ± 0.28	19.31 ± 0.21	20.69 ± 0.19
Ferritin	22.68 ± 0.37	21.17 ± 0.14	20.05 ± 0.13	20.75 ± 0.32	19.49 ± 0.05	21.21 ± 0.22
GAPDH	21.70 ± 0.23	19.65 ± 0.13	18.58 ± 0.08	19.80 ± 0.44	18.20 ± 0.15	20.03 ± 0.23
α-TUB	26.96 ± 0.23	25.54 ± 0.32	20.79 ± 0.21	25.11 ± 1.33	21.04 ± 0.27	24.69 ± 0.46
β-TUB	23.92 ± 0.16	23.88 ± 0.48	22.05 ± 0.22	21.24 ± 0.16	21.38 ± 0.17	22.38 ± 0.21

AK	21.08 ± 0.35	20.82 ± 0.15	18.95 ± 0.14	21.41 ± 0.76	18.77 ± 0.06	20.50 ± 0.25
GST	21.65 ± 0.39	20.41 ± 0.31	20.57 ± 0.30	20.62 ± 0.31	19.55 ± 0.07	20.77 ± 0.19
RPL8	20.36 ± 0.22	20.32 ± 0.14	19.03 ± 0.05	19.50 ± 0.34	18.17 ± 0.07	19.71 ± 0.16
RPL10	18.22 ± 0.41	19.08 ± 0.18	19.83 ± 0.14	20.20 ± 0.18	19.87 ± 0.15	19.22 ± 0.19
RPL32	19.44 ± 0.31	20.98 ± 0.11	28.10 ± 0.57	31.44 ± 0.49	22.35 ± 0.09	23.69 ± 0.72
RPS11	20.86 ± 0.59	20.89 ± 0.16	20.05 ± 0.34	20.21 ± 0.23	19.40 ± 0.10	20.43 ± 0.23
RPS15	19.80 ± 0.36	18.96 ± 0.17	20.06 ± 0.21	20.10 ± 0.34	18.88 ± 0.04	19.60 ± 0.16
RPS20	22.10 ± 0.63	21.50 ± 0.29	19.84 ± 0.27	21.06 ± 0.28	20.56 ± 0.06	21.27 ± 0.26

Note: mean±*SE*.

Table S3. Expression stability ranking of 15 candidate RGs was calculated using Delta Ct, GeNorm and NormFinder and BestKeeper under five different insecticide stresses. The average expression stability (M value,) is listed by Delta Ct, GeNorm and NormFinder analysis, and standard deviation (SD) and coefficient of variation (CV) were given by BestKeeper analysis (the lower, the most stable). The stability decreased from top to bottom.

Rank	Delta Ct method		NormFinder		Genorm		BestKeeper		
	Gene	M	Gene	M	Gene	M	Gene	SD	CV
Imidacloprid									

1	*GST*	0.07	*AK*	0.00	*AK*	0.00	*RPL10*	0.01	0.04
2	*RPS15*	0.07	*RPL10*	0.00	*RPL10*	0.00	*AK*	0.01	0.04
3	*RPL10*	0.07	*RPS15*	0.00	*RPS15*	0.00	*RPS15*	0.01	0.06
4	*AK*	0.07	*GST*	0.01	*GST*	0.01	*GAPDH*	0.02	0.07
5	*GAPDH*	0.07	*GAPDH*	0.02	*GAPDH*	0.02	*GST*	0.02	0.09
6	*RPS20*	0.09	*RPS20*	0.04	*RPS20*	0.04	*α-TUB*	0.03	0.10
7	*EF2*	0.09	*EF2*	0.05	*EF2*	0.05	*EF2*	0.03	0.16
8	*EF1α*	0.09	*EF1α*	0.05	*EF1α*	0.05	*EF1α*	0.03	0.17
9	*α-TUB*	0.10	*α-TUB*	0.05	*α-TUB*	0.05	*RPS20*	0.05	0.25
10	*RPL8*	0.12	*RPL8*	0.10	*RPL8*	0.10	*Ferritin*	0.08	0.39
11	*RPS11*	0.12	*RPS11*	0.10	*RPS11*	0.10	*RPL8*	0.09	0.45
12	*β-TUB*	0.15	*Ferritin*	0.13	*Ferritin*	0.13	*RPS11*	0.09	0.45
13	*Ferritin*	0.15	*β-TUB*	0.14	*β-TUB*	0.14	*β-TUB*	0.11	0.49
14	*RPL32*	0.16	*RPL32*	0.14	*RPL32*	0.14	*RPL32*	0.11	0.58
15	*β-actin*	0.20	*β-actin*	0.19	*β-actin*	0.19	*β-actin*	0.12	0.61
**Thiamethoxam**									
1	*RPL10*	0.09	*RPL10*	0.01	*GAPDH*	0.01	*AK*	0.01	0.03
2	*RPS11*	0.09	*AK*	0.02	|*RPS15*	0.01	*RPL10*	0.01	0.07

3	*AK*	0.09	*RPS11*	0.02	*RPS11*	0.01	*RPS11*	0.01	0.07
4	*RPS15*	0.10	*RPS15*	0.04	*AK*	0.02	*GAPDH*	0.02	0.10
5	*GAPDH*	0.10	*GAPDH*	0.05	*RPL10*	0.03	*RPS15*	0.02	0.10
6	*β-actin*	0.10	*β-actin*	0.05	*EF1α*	0.03	*EF1α*	0.03	0.15
7	*EF1α*	0.11	*EF1α*	0.07	*β-actin*	0.05	*β-actin*	0.05	0.25
8	*RPS20*	0.12	*RPS20*	0.08	*β-TUB*	0.05	*β-TUB*	0.06	0.24
9	*β-TUB*	0.12	*β-TUB*	0.08	*RPS20*	0.07	*GST*	0.07	0.34
10	*GST*	0.13	*GST*	0.09	*GST*	0.08	*RPS20*	0.08	0.40
11	*RPL32*	0.15	*RPL32*	0.14	*RPL32*	0.09	*EF2*	0.09	0.42
12	*EF2*	0.16	*EF2*	0.14	*RPL8*	0.10	*RPL8*	0.11	0.57
13	*RPL8*	0.16	*RPL8*	0.15	*α-TUB*	0.11	*Ferritin*	0.12	0.55
14	*α-TUB*	0.17	*α-TUB*	0.16	*EF2*	0.12	*RPL32*	0.12	0.66
15	*Ferritin*	0.20	*Ferritin*	0.20	*Ferritin*	0.13	*α-TUB*	0.14	0.51
**λ-cyhalothrin**									
1	*RPL10*	0.05	*RPL32*	0.01	*RPL10*	0.01	*EF1α*	0.00	0.02
2	*RPL32*	0.05	*RPS11*	0.01	*RPL32*	0.01	*RPL32*	0.01	0.05
3	*RPS11*	0.05	*RPL10*	0.02	*EF2*	0.02	*EF2*	0.01	0.06
4	*EF1α*	0.05	*Ferritin*	0.02	*EF1α*	0.02	*Ferritin*	0.01	0.06
5	*Ferritin*	0.05	*RPS15*	0.02	*Ferritin*	0.02	*RPL10*	0.02	0.08

6	*RPL8*	0.05	*RPL8*	0.02	*RPS11*	0.03	*RPS11*	0.02	0.09
7	*EF2*	0.05	*EF1α*	0.02	*RPL8*	0.03	*RPS20*	0.03	0.11
8	*RPS15*	0.06	*EF2*	0.03	*RPS15*	0.03	*AK*	0.03	0.13
9	*β-actin*	0.06	*β-actin*	0.03	*β-actin*	0.03	*RPL8*	0.03	0.14
10	*AK*	0.07	*AK*	0.04	*AK*	0.04	*β-actin*	0.03	0.15
11	*RPS20*	0.07	*RPS20*	0.05	*RPS20*	0.04	*RPS15*	0.03	0.15
12	*GST*	0.08	*GST*	0.06	*GST*	0.04	*GST*	0.05	0.21
13	*β-TUB*	0.08	*β-TUB*	0.07	*β-TUB*	0.05	*α-TUB*	0.06	0.22
14	*α-TUB*	0.11	*α-TUB*	0.11	*α-TUB*	0.06	*β-TUB*	0.06	0.23
15	*GAPDH*	0.17	*GAPDH*	0.17	*GAPDH*	0.07	*GAPDH*	0.13	0.57
**Abamectin**									
1	*RPL10*	0.07	*RPS15*	0.01	*GAPDH*	0.01	*RPS15*	0.01	0.05
2	*RPS15*	0.07	*RPL10*	0.01	*RPL10*	0.01	*RPL10*	0.01	0.06
3	*GAPDH*	0.08	*EF1α*	0.02	*RPS15*	0.02	*GAPDH*	0.01	0.06
4	*RPL32*	0.08	*GAPDH*	0.02	*RPL32*	0.02	*RPL32*	0.02	0.09
5	*EF1α*	0.08	*RPL32*	0.03	*RPS11*	0.03	*EF1α*	0.02	0.11
6	*EF2*	0.09	*EF2*	0.04	*EF1α*	0.03	*RPS11*	0.03	0.16
7	*RPS11*	0.09	*RPS11*	0.06	*Ferritin*	0.04	*EF2*	0.04	0.19
8	*GST*	0.09	*GST*	0.06	*EF2*	0.05	*Ferritin*	0.05	0.23

9	*Ferritin*	0.10	*Ferritin*	0.08	*GST*	0.06	*GST*	0.05	0.24
10	*AK*	0.11	*AK*	0.09	*β-actin*	0.06	*β-actin*	0.06	0.33
11	*β-actin*	0.11	*β-actin*	0.09	*RPL8*	0.07	*AK*	0.07	0.36
12	*RPL8*	0.13	*RPL8*	0.11	*AK*	0.08	*RPL8*	0.08	0.39
13	*α-TUB*	0.13	*α-TUB*	0.12	*α-TUB*	0.09	*α-TUB*	0.09	0.35
14	*β-TUB*	0.15	*β-TUB*	0.14	*β-TUB*	0.09	*β-TUB*	0.10	0.44
15	*RPS20*	0.17	*RPS20*	0.16	*RPS20*	0.10	*RPS20*	0.11	0.54
**Matrine**									
1	*RPL10*	0.09	*RPL8*	0.01	*GST*	0.01	*RPL10*	0.01	0.06
2	*RPL8*	0.09	*RPL10*	0.01	*AK*	0.01	*RPL8*	0.01	0.06
3	*GAPDH*	0.10	*GAPDH*	0.03	*RPL8*	0.03	*AK*	0.03	0.13
4	*AK*	0.10	*AK*	0.04	*RPL10*	0.03	*GAPDH*	0.03	0.14
5	*GST*	0.10	*GST*	0.05	*Ferritin*	0.05	*GST*	0.04	0.16
6	*β-actin*	0.10	*β-actin*	0.05	*EF1α*	0.05	*RPS15*	0.04	0.16
7	*RPS15*	0.10	*RPS15*	0.05	*RPS15*	0.06	*β-actin*	0.05	0.20
8	*EF2*	0.11	*EF2*	0.07	*GAPDH*	0.07	*EF2*	0.05	0.23
9	*RPS20*	0.12	*RPS20*	0.08	*β-actin*	0.07	*RPS20*	0.06	0.24
10	*Ferritin*	0.13	*Ferritin*	0.10	*EF2*	0.08	*EF1α*	0.07	0.32
11	*EF1α*	0.13	*EF1α*	0.10	*RPS20*	0.08	*Ferritin*	0.07	0.26

12	*α-TUB*	0.15	*α-TUB*	0.12	*RPL32*	0.09	*RPL32*	0.08	0.40
13	*RPL32*	0.15	*RPL32*	0.12	*α-TUB*	0.10	*α-TUB*	0.09	0.31
14	*EF2*	0.65	*RPS11*	0.18	*RPS11*	0.11	*RPS11*	0.11	0.46
15	*α-TUB*	0.84	*β-TUB*	0.22	*β-TUB*	0.13	*β-TUB*	0.16	0.66

Table S4. Comprehensive gene stability ranking of 15 candidate reference genes in all samples was calculated using RefFinder. The Genes Geomean of ranking values is listed, and the stability decreased from top to bottom.

Rank	Insecticide treatment		Temperatures		Odor stimulation		Developmental stages		Both sexes		All samples	
	**Gene**	**GM**	**Gene**	**GM**	**Gene**	**GM**	**Gene**	**GM**	**Gene**	**GM**	**Gene**	**GM**
1	*EF2*	2.21	*EF1a*	1.73	*EF1a*	2.34	*Actin*	1.5	*Actin*	1.5	*EF2*	2
2	*RPL32*	3.6	*Actin*	2.21	*RPL8*	3.16	*EF2*	2	*RPS15*	2.63	*RPL8*	2.78
3	*GAPDH*	3.98	*RPL32*	2.83	*Ferritin*	3.44	*RPS15*	4.36	*RPL8*	3.64	*GST*	2.94
4	*EF1a*	5.03	*RPL8*	3.94	*Actin*	4.03	*GST*	4.56	*Ferritin*	3.71	*Ferritin*	3.66
5	*GST*	5.07	*Ferritin*	4.14	*EF2*	4.16	*RPS11*	5.01	*AK-1*	3.98	*GAPDH*	4.03
6	*a-TUB*	5.18	*EF2*	4.7	*a-TUB*	5.2	*RPS20*	5.48	*RPS20*	4.76	*RPS15*	4.53
7	*RPS15*	5.18	*RPL10*	6.82	*GAPDH*	5.58	*β-TUB*	6.45	*GST*	6.05	*EF1a*	7
8	*RPL8*	5.90	*GAPDH*	7.14	*RPS15*	6.48	*RPL10*	7.01	*RPL32*	6.82	*RPS11*	7.71
9	*AK-1*	6.12	*AK-1*	8.61	*RPL10*	7.2	*Ferritin*	7.48	*EF1a*	7.75	*RPL10*	9.01
10	*β-TUB*	6.85	*RPS11*	9.43	*AK-1*	7.2	*EF1a*	7.97	*RPS11*	9.49	*RPS20*	9.39
11	*Ferritin*	8.68	*RPS15*	10.22	*β-TUB*	8.24	*GAPDH*	9.43	*GAPDH*	11	*AK-1*	9.43
12	*RPL10*	9.19	*RPS20*	12	*GST*	11.98	*RPL8*	9.72	*RPL10*	12	*Actin*	10.47
13	*Actin*	12.24	*a-TUB*	13.24	*RPS11*	13.24	*AK-1*	13.24	*β-TUB*	13	*β-TUB*	10.61
14	*RPS11*	14	*GST*	13.74	*RPS20*	13.47	*RPL32*	13.74	*EF2*	14	*a-TUB*	14
15	*RPS20*	15	*β-TUB*	15	*RPL32*	15	*a-TUB*	15	*a-TUB*	15	*RPL32*	15

Rank	Imidacloprid		Thiamethoxam		λ-cyhalothrin		Abamectin		Matrine	
	**Gene**	**GM**	**Gene**	**GM**	**Gene**	**GM**	**Gene**	**GM**	**Gene**	**GM**
1	*RPL10*	1.57	*RPL10*	1.78	*RPL32*	1.19	*RPS15*	1.32	*RPL10*	1.68
2	*RPS15*	2.06	*AK-1*	2.21	*RPL10*	2.34	*RPL10*	1.68	*RPL8*	1.86
3	*AK-1*	2.21	*RPS11*	2.71	*EF1a*	3.25	*GAPDH*	2.45	*AK-1*	2.83
4	*GST*	2.99	*RPS15*	2.83	*RPS11*	3.83	*RPL32*	4.23	*GST*	3.5
5	*GAPDH*	4.73	*GAPDH*	3.34	*Ferritin*	4.16	*EF1a*	4.61	*GAPDH*	3.83
6	*EF2*	6.74	*EF1a*	6.48	*EF2*	5.09	*RPS11*	6.19	*RPS15*	6.44
7	*RPS20*	7.14	*Actin*	6.48	*RPL8*	6.9	*EF2*	6.7	*Actin*	6.9
8	*EF1a*	7.74	*β-TUB*	8.49	*RPS15*	7.52	*GST*	8.24	*Ferritin*	8.41
9	*a-TUB*	8.13	*RPS20*	8.71	*AK-1*	9.46	*Ferritin*	8.45	*EF2*	8.46
10	*RPL8*	10.72	*GST*	9.74	*Actin*	9.46	*Actin*	10.49	*EF1a*	9.45
11	*RPS11*	10.74	*RPL32*	11.68	*RPS20*	9.82	*AK-1*	10.72	*RPS20*	9.46
12	*Ferritin*	11.93	*EF2*	12.2	*GST*	12	*RPL8*	11.74	*RPL32*	12.49
13	*β-TUB*	12.72	*RPL8*	12.49	*β-TUB*	13	*a-TUB*	13	*a-TUB*	12.49
14	*RPL32*	13.74	*a-TUB*	13.98	*a-TUB*	14	*β-TUB*	14	*RPS11*	14
15	*Actin*	15	*Ferritin*	14.47	*GAPDH*	15	*RPS20*	15	*β-TUB*	15

Note: GM, geometric mean

Table S5. FPKM values of *B. gobica* nymphs and adults before and after imidacloprid treatment in the transcriptome data. FPKM: fragments per kilobase of exon model per million mapped reads.

Sample names	FPKM values		
	**Repeat 1**	**Repeat 2**	**Repeat 3**
Nymphs without imidacloprid treatment	2.86	2.11	0.24
Nymphs with imidacloprid treatment	0.90	1.50	1.74

Adults without imidacloprid treatment	11.43	7.10	11.77

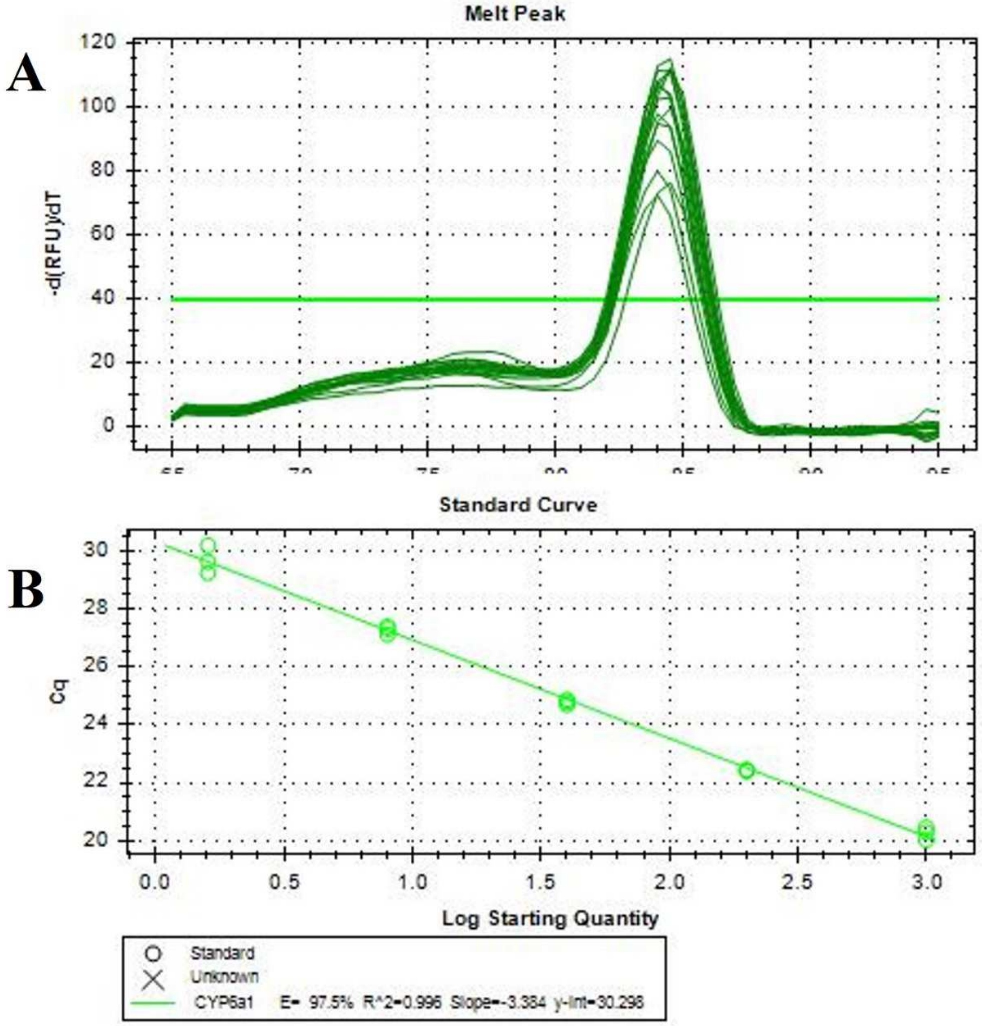

Figure S1. Specificity and amplification efficiency of primer pairs of target gene *CYP6a1* by RT-qPCR in *B. gobica*. A: The melt peaks of primers of *CYP6a1*. B: The standard curve of primers of *CYP6a1*.
